# Development of an Integrated Surveillance System to Improve Preparedness for Arbovirus Outbreaks in a Dengue Endemic Setting: Descriptive Study

**DOI:** 10.2196/62759

**Published:** 2024-11-14

**Authors:** André Leandro, Rafael Maciel-de-Freitas

**Affiliations:** 1 Centro de Controle de Zoonoses, Secretaria Municipal de Saúde de Foz do Iguaçu, Foz do Iguaçu, Brazil; 2 Laboratório de Mosquitos Transmissores de Hematozoários, Instituto Oswaldo Cruz, Fiocruz, Rio de Janeiro, Brazil; 3 Department of Arbovirology, Bernhard Nocht Institute for Tropical Medicine, Bernhard Nocht Straße 74, Hamburg, 20359, Germany, 49 40 2853800

**Keywords:** surveillance, Aedes aegypti, vector control, transmission risk, dengue fever

## Abstract

**Background:**

Dengue fever, transmitted by *Aedes aegypti* and *Aedes albopictus* mosquitoes, poses a significant public health challenge in tropical and subtropical regions. Dengue surveillance involves monitoring the incidence, distribution, and trends of infections through systematic data collection, analysis, interpretation, and dissemination. It supports public health decision-making, guiding interventions like vector control, vaccination campaigns, and public education.

**Objective:**

Herein, we report the development of a surveillance system already in use to support public health managers against dengue transmission in Foz do Iguaçu, a dengue-endemic Brazilian city located in the Triple Border with Argentina and Paraguay.

**Methods:**

We present data encompassing the fieldwork organization of more than 100 health agents; epidemiological and entomological data were gathered from November 2022 to April 2024, totalizing 18 months of data collection.

**Results:**

By registering health agents, we were able to provide support for those facing issues to fill their daily milestone of inspecting 16 traps per working day. We filtered dengue transmission in the city by patient age, gender, and reporting units, as well as according to dengue virus serotype. The entomological indices presented a strong seasonal pattern, as expected. Several longtime established routines in Foz do Iguaçu have been directly impacted by the adoption of Vigilância Integrada com Tecnologia (VITEC).

**Conclusions:**

The implementation of VITEC has enabled more efficient and accurate diagnostics of local transmission risk, leading to a better understanding of operational activity patterns and risks. Lately, local public health managers can easily identify hot spots of dengue transmission and optimize interventions toward those highly sensitive areas.

## Introduction

Dengue fever, a mosquito-borne viral disease, poses a significant public health challenge in many tropical and subtropical regions worldwide [1]. Caused by 4 antigenically distinct serotypes, the dengue virus (DENV) is transmitted primarily by the bite of *Aedes aegypti* and *Aedes albopictus* mosquitoes. Dengue infection can lead to severe flu-like symptoms and, in severe cases, life-threatening complications such as dengue hemorrhagic fever and dengue shock syndrome [2-4]. The global burden of dengue has escalated dramatically over recent decades, driven by factors such as unorganized urbanization, climate change, increased international travel, lack of effective and timely interventions, and spread insecticide resistance of native vector populations [5-9]. As a result, effective dengue surveillance systems are crucial for early detection, timely response, and the prevention of outbreaks [10-14].

Dengue surveillance encompasses a range of activities aimed at monitoring the incidence, distribution, and trends of dengue infections. These activities include the systematic collection, analysis, interpretation, and dissemination of data related to dengue cases and vector populations [12,14-20]. Surveillance systems provide critical information that supports public health decision-making, guiding interventions such as vector control, vaccination campaigns, and public education efforts. Moreover, robust surveillance enables the assessment of intervention efficacy and facilitates the timely allocation of resources during outbreaks [21-24].

The actual epidemiological surveillance in most endemic countries in Latin America has a strong reactive component, which takes place after screening symptomatic persons who seek care in the health system in cities [24]. Entomological surveillance is often based on conducting larval surveys in a randomly selected fraction of the urban area within cities each cycle. The selected area corresponds to 2%‐10% of dwellings and aims to reveal the diversity of available breeding sites and trigger further targeting of the most productive breeding sites [25-27]. However, this approach has limited efficacy in determining the areas within cities at higher risk of dengue transmission due to a low correlation between larval surveys and immature sampling with dengue transmission [22,25,28]. Therefore, dengue surveillance could be enhanced to incorporate more effective approaches in both epidemiological and entomological components.

Effective dengue surveillance requires an integrated approach, combining epidemiological, entomological, and laboratory data. Epidemiological surveillance could be enhanced by tracking human cases to identify patterns and potential hot spots of transmission [29,30]. Moreover, entomological surveillance could be improved by promoting citywide trapping focused on collecting the adult mosquito population to provide reliable and timely insights into vector density and distribution [22,23,31,32]. Furthermore, laboratory surveillance, including rapid serological and molecular diagnostics on both field-gathered mosquitoes and humans, aids in confirming cases and identifying circulating DENV serotypes [33-36]. One key challenge to promoting an integrated surveillance approach involves working with complex datasets from different sources. Large metropolitan areas such as dengue endemic regions in Latin America and Southeast Asia, have enormous data banks that make the extraction of useful surveillance information to support further intervention in a timely manner to improve the response to dengue outbreaks a tough challenge.

In summary, dengue surveillance is a cornerstone of dengue control and prevention efforts, providing the essential data needed to mitigate the impact of this pervasive disease [11]. By fostering early detection and informed intervention strategies, surveillance systems play a vital role in safeguarding public health and reducing the burden of dengue worldwide [37]. In this report, we share the development of a web-based and portable surveillance system to gather epidemiological, entomological, and demographic data to create an integrated dengue surveillance system to support local public health managers. We also present results gathered at the city of Foz do Iguaçu to highlight how this system supports public health managers and vector control personnel working with dengue surveillance.

## Methods

### Study Area

The study was conducted in the city of Foz do Iguaçu (25°30′58″ S, 54°35′07″ W), Brazil, a prominent tourist destination located at the Triple Border with Paraguay and Argentina. The city is geographically distinct, bordered by the Paraná River to the west, the Itaipu Hydroelectric Power Plant to the north, and the Iguazu National Park to the south. Despite this relative isolation, there is significant movement between Brazil and Paraguay, primarily driven by trade and smuggling activities. Foz do Iguaçu has a population of approximately 260,000 residents, distributed across 73 urban areas with around 1500 premises each. The climate in Foz do Iguaçu is classified as humid tropical according to the Köppen–Geiger system. The region experiences hot and humid summers, with mean temperatures exceeding 27 °C, and mild to cold winters, with mean temperatures around 17 °C. The average annual rainfall is approximately 1850 mm. Since the 1990s, Foz do Iguaçu has been endemic for dengue transmission, experiencing outbreaks every 4‐5 years. This persistent pattern of dengue outbreaks underscores the necessity for ongoing surveillance and public health interventions to mitigate the impact of the disease.

### Surveillance System

VITEC is an acronym for Vigilância Integrada com Tecnologia (Integrated Surveillance with Technology). VITEC is an integrated surveillance software with information technology for the Public Health area to manage and automate programs aimed at vector-borne diseases and other zoonoses. It was developed with the aim of enabling public health managers to integrate entomological and epidemiological indicators into a single software program in order to define prevention and vector control field actions based on transmission risk scenarios. It promotes the empowerment of the health manager and generates automated reports to support public health managers in their routine decision-making. Herein we present an example of VITEC’s usage for gathering and integrating demographic, entomological, and epidemiological data regarding arbovirus transmission in the city of Foz do Iguaçu. Due to the disproportional number of cases, our main interest is in DENV, although some cases of both Zika and chikungunya were also reported in the city. Our dataset comprises 18 months, from November 2022 to April 2024. VITEC enrolled 100 health agents from Foz do Iguaçu in October 2022.

### Collecting and Organizing Data (Ladder Model)

A critical step for developing the VITEC involved systematizing the data collection and organizing it according to a ladder model. The step-by-step decision-making process commences with the systematic collection of data, ensuring the inclusion of essential information pertinent to subsequent stages while avoiding both the omission of critical data and the unnecessary accumulation of superfluous information. The selection of data to be collected, along with the methodologies for its collection and storage, are pivotal aspects in constructing an effective decision-making framework. In our study, we used VITEC as the technological tool for data collection, which facilitated the answering of fundamental surveillance questions (where, when, and who) by segmenting the data into distinct units: (1) spatial (property); (2) temporal (date, hour, or minute); and (3) population (individual). Efficient and accurate data collection enabled a seamless transition to the subsequent step of generating reports for information analysis. As the process progressed, we moved naturally through the stages, including the selection of priorities based on the produced reports, thereby enabling the formulation of control strategies and the implementation of measures to prevent or mitigate transmission. This process continued iteratively, with ongoing data collection to monitor and evaluate the outcomes of the implemented control measures. Given the substantial volume of data and the rapid progression associated with disease spread, outbreaks, and epidemics, it became imperative to seek advanced software and equipment to modernize work processes. This modernization spanned from data collection by health agents using mobile computing devices to integrated surveillance systems for information analysis and priority setting, thereby characterizing risk scenarios related to arboviruses. VITEC is an integrated system that uses georeferenced data, facilitating the creation of maps, graphs, and tables accessible via the internet. This capability allows for real-time risk characterization at an “infra-municipal” level, significantly enhancing our ability to manage and respond to disease risks effectively.

### Organizing Operational Data (Human Resources)

The organization of services is a critical factor in achieving successful outcomes. To configure services within a replica of the system, a local administrative manager must first create a login and password to gain access and initiate the process of enabling the municipality in the web environment. The initial step involves establishing the territorial base by importing map files of the municipal territories or creating maps using the available map creation tool. Subsequently, the administrator registers employees (field coordinators, field supervisors, and health agents) and assigns their respective logins and passwords. Employees may be organized into teams based on operational requirements. Registration is conducted hierarchically, with access to the system’s functions and screens granted according to each employee’s hierarchical level. Following this, the administrator can plan activities and generate work orders, referred to as demands, which are then assigned to the registered employees or teams. Each employee downloads the application for data collection from the Google Play Store. Using their login credentials, employees can access and synchronize data, thereby retrieving the work orders issued via the web platform. Upon completing the work orders using the predefined forms within the application, the employee synchronizes the data, which involves sending collected data to the database and receiving updates from the database. This synchronization occurs via internet access, although the application is designed to function both online and offline. It captures information from the forms, geographic coordinates (latitude and longitude), and timestamps for task execution. The application also records samples collected in the field, which are then sent to the laboratory for screening. Once the diagnoses are complete, the results are entered into a web-based environment designed for recording diagnostic data.

### Obtaining Epidemiological Data (Information System for Notifiable Diseases)

The health care system in Foz do Iguaçu is evenly distributed throughout the city and currently comprises 36 health units, from clinics to hospitals [38]. The Ministry of Health classifies dengue as a notifiable disease, mandating that cases be reported at any local health facility. Epidemiological data are recorded in the Information System for Notifiable Diseases (SINAN), accessible to public health teams in all Brazilian cities. The decentralization and modernization of epidemiological surveillance in Foz do Iguaçu started in 2009 by providing conditions and personnel training in each of the city health units to conduct the dengue notification in the locus. Health care network professionals, including approximately 500 community health agents affiliated with the Basic Health Units and 150 endemic disease control agents from the zoonosis surveillance unit, received training to conduct epidemiological surveillance activities. They were encouraged to actively search for symptomatic cases and refer these cases to health units for assistance and notification. A suspected dengue case was reported when a resident of Foz do Iguaçu exhibited at least 1 symptom compatible with dengue, including fever, headache, myalgia, arthralgia, rash, nausea, retro-orbital pain, petechiae, or malaise, within the preceding 14 days, in accordance with the National Guidelines from the Brazilian Ministry of Health [38]. Before the adoption of VITEC, 1 single person in the epidemiology section from Foz do Iguaçu was responsible for downloading data from SINAN, analyze and present to the entomology or vector control team on a fortnightly basis. The data collected via SINAN at the notifying units are subsequently imported into VITEC, which integrates this information with other indicators for comprehensive risk assessment and decision-making. A tool developed within VITEC facilitates the import and integration of files exported from SINAN into a database. The imported data are processed by an algorithm using a paid Google API, resulting in the generation of georeferenced reports. This mechanism enables the frequent and continuous updating of epidemiological information.

### Collecting Entomological Data (Adultraps)

The *A aegypti* population is monitored in Foz do Iguaçu using 2 distinct traps, each one sampling a specific stage of the mosquito life cycle. For capturing adult mosquitoes, both males and females, we used Adultraps [39,40], whereas *A aegypti* eggs were sampled using ovitraps [41]. Adultraps are specifically designed to capture gravid *A aegypti* females during oviposition, using water as the primary attractant. The trap features an opening at the top through which the mosquitoes enter and are subsequently trapped in an interior chamber. The water, contained in a compartment at the bottom, is inaccessible to the mosquitoes, thus preventing egg-laying [39,40]. The sampling was performed in 12 areas of 1 km^2^ each, homogeneously distributed across the city [42]. Those 12 areas present similar variation in entomological indexes as in larger areas; therefore, they represent an optimization of sampling. In each of those areas, 25 Adultraps and 25 ovitraps are evenly distributed and inspected weekly. Therefore, considering our 18-month evaluation and 300 traps inspected per week, our study period comprised a total of 74 weeks and approximately 22,200 trap inspections. Previous analyses of data collected in Foz do Iguaçu have demonstrated that entomological indices based on adult *A aegypti* sampling are more effective in predicting dengue outbreaks 4 weeks in advance compared to traditional indices such as the House Index and the Breteau Index [22,38,42]. Among the adult-based indices, 2 have shown superior performance in forecasting dengue outbreaks—the Trap Positivity Index (TPI) and the Adult Density Index (ADI). The TPI is defined as the number of positive traps among the total number of traps inspected, multiplied by 100. The ADI is calculated as the total number of *A aegypti* mosquitoes captured divided by the total number of inspected traps, multiplied by 100 [22]. These indices were selected for further analysis due to their enhanced predictive capabilities.

### Collecting Entomological Data (Ovitraps)

This trap is specifically designed for oviposition and does not collect adult mosquitoes. Two entomological indices are calculated based on the collected eggs: (1) Ovitrap Positivity Index (OPI), which consists of the percentage of traps with at least 1 egg collected, and (2) Egg Density Index (EDI), which represents the average number of eggs considering the positive ovitraps [25]. A total of 8 employees and 4 vehicles inspected these traps weekly, and each employee has a daily target of inspecting 16 mosquito traps. The traps are installed in the field using the VITEC application. Each trap is assigned a unique identifier using QR codes. Scanning the QR code through the application or manually entering the trap number georeferenced the trap, capturing latitude and longitude without the need for mobile data connectivity. Once installed, pertinent information about each trap is recorded to facilitate access during future surveys and to link data within the database. Samples collected in the field are recorded in the VITEC mobile app, including their geographic coordinates (latitude and longitude). The collected materials, comprising adult mosquitoes and eggs in straws, are sent to the laboratory. There, mosquitoes are counted and classified, and eggs are counted by an employee using a stereoscopic microscope. VITEC provides an environment for recording laboratory results, which is integrated with all system environments and the mobile app. The system uses these data for automated index calculations, which are subsequently presented as maps and graphs.

### Ethical Considerations

According to the Brazilian federal legislation (Portaria de Consolidação GM/MS n° 4, September 28, 2017), dengue is one of the diseases of compulsory notification in the country. Therefore, all medical staff working in the 48,161 public health units and 4466 private hospitals distributed across the country must report disease occurrence. By corollary, the original data collection did not require institutional review board approval. The work presented herein used only secondary data. The Ministry of Health legislation (Law 66, from December 10, 2004) establishes the procedures and responsibilities relating to the technical-scientific dissemination of data and information from the Health Surveillance Secretariat—Secretaria de Vigilância em Saúde/Ministério da Saúde. Since the coauthor AL is a civil servant, one of the public health managers of the city of Foz do Iguaçu, among his professional routine duties are accessing and analyzing disease occurrence data within the city he works for. Thus, permission to access secondary data regarding disease occurrence for surveillance purposes was granted to coauthor AL. VITEC respects the general data protection laws and preserves the identity of each patient, not exposing addresses or individual characteristics. The reports available are produced at levels of spatial aggregation sufficient for decision-making while preserving the confidentiality of the information.

## Results

### Organizing Operational Data (Human Resources)

**Figure 1.**
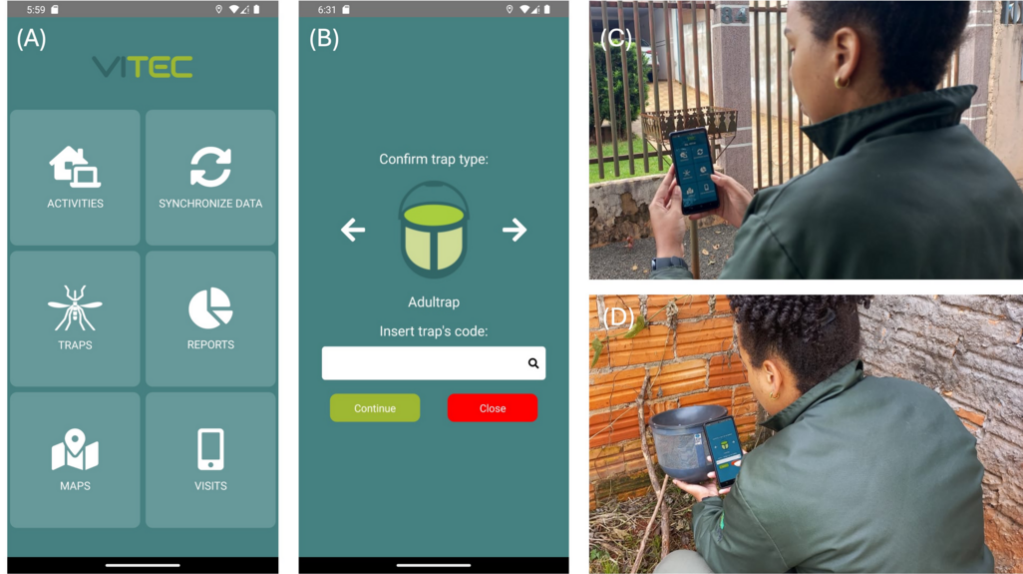
Routine of field surveillance in the city of Foz do Iguaçu. (**A**) The app home screen showing the interactive menu; (**B**) screen to inform data for Adultrap installment in the field; (**C**) cataloging 1 house to receive 1 Adultrap; and (**D**) reading the QR code during weekly trap inspection.

A total of 110 employees were registered in Foz do Iguaçu, comprising 1 general system administrator, 9 service coordinators, and 100 field operation agents. An example of a routine service is available in Figure 1. For both routine services and situations identified as having evident entomo-epidemiological risk within the system, appropriate actions were planned. The adoption of VITEC by the Foz do Iguaçu zoonosis control team significantly reduced the time required for the case report to become available for the vector control. If before VITEC, the dengue incidence in the city was downloaded and analyzed by a single person that reported the epidemiological cases to the vector control fortnightly; this now is done daily. Furthermore, the VITEC automation produces the epidemiological curve of the city in less than 2 minutes, giving all the conditions for the vector control team to promote interventions in the most sensitive areas. These interventions included routine services and transmission-blocking measures such as ultralow volume fogging. The generated work orders were assigned to employees, who then executed them in the field using the mobile app.

The health agents from Foz do Iguaçu have a daily goal to inspect 16 mosquito traps. After the adoption of VITEC, the local public health manager estimated the health agents had an average of 12 traps inspected per day. The time spent in each house for inspecting each trap was available and one of the less productive health agents was identified, as well as the time he started working and the time spent per house (Multimedia Appendix 1). With such information available, the field team supervisor was able to facilitate the support necessary for the reestablishment of activities, with the objective of achieving alignment with the agreed target.

### Gathering and Analyzing Epidemiological Data

**Figure 2.**
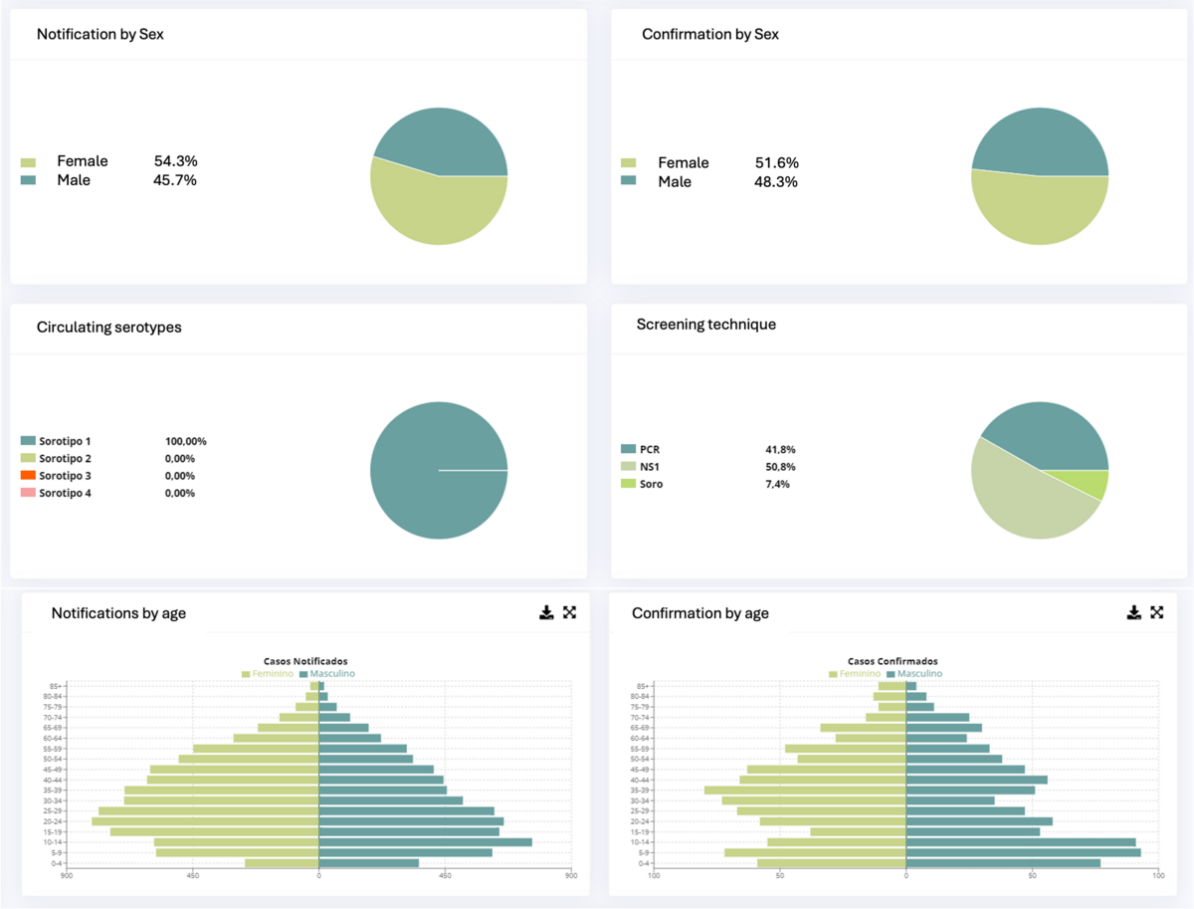
Epidemiological dashboard showing the notification and confirmation of dengue cases in 2023 by sex, age, serotype, and the screening technique used. The panel is composed of figures extracted from VITEC. VITEC: Vigilância Integrada com Tecnologia.

**Figure 3.**
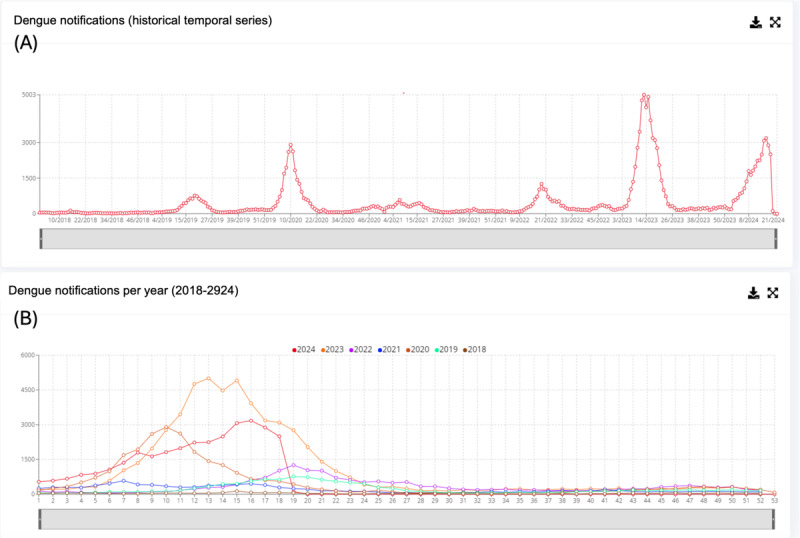
Epidemiological dashboard from Foz do Iguaçu. (**A**) The historical temporal series of DENV notifications In Foz do Iguaçu since VITEC was developed. (**B**) Dengue notifications per year. The panel is composed of figures extracted from VITEC. DENV: dengue virus; VITEC: Vigilância Integrada com Tecnologia.

Data from SINAN were imported daily into VITEC. Once imported, VITEC automatically generates reports on suspected and confirmed cases, enabling a spatiotemporal analysis of disease incidence (Figure 2). These reports include distributions by neighborhood, age group, gender, and reporting units, among other analytical categories (Figure 2). Since SINAN only provides raw data, that is, does not generate reports for decision-making purposes, the automation of results by VITEC significantly enhances the speed of result availability. This automation allows for tracking the temporal evolution of disease cases within the analyzed period and facilitates the importation of historical data from previous years. Consequently, the system can highlight the marked seasonality of the disease and enable comparisons between the current period and the past years (Figure 3).

### Gathering and Analyzing Entomological Data (Adultrap and Ovitrap)

**Figure 4.**
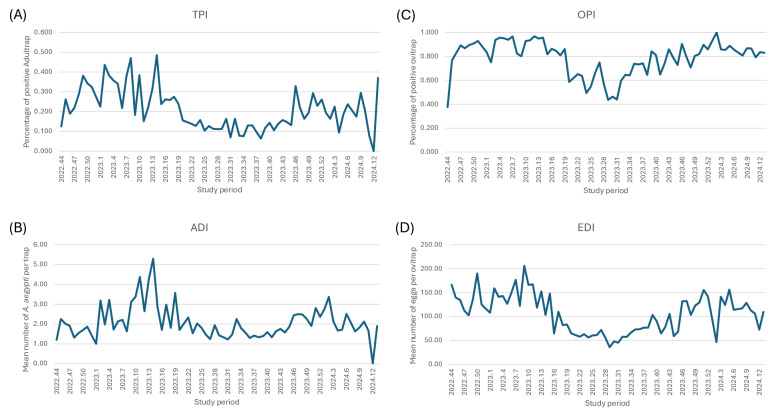
Entomological indices obtained from Foz do Iguaçu during November 2022 to April 2024. (**A**) TPI: the percentage of positive Adultraps; (**B**) ADI: average number of adult *Aedes aegypti* per Adultrap; (**C**) OPI: the percentage of positive ovitraps; and (**D**) EDI: average number of eggs per ovitrap. ADI: Adult Density Index; EDI: Egg Density Index; OPI: Ovitrap Positivity Index; TPI: Trap Positivity Index.

A total of 300 Adultrap and 300 ovitraps were installed and georeferenced within 12 sentinel geographical units, which were monitored weekly over the course of 74 epidemiological weeks. The samples collected from these traps were analyzed in the VITEC laboratory, resulting in automated reports that generated infestation indices. These indices were visualized in the form of graphs and maps, spatially representing the risk of disease transmission. The weekly analysis of entomological indices produced by the 2 types of traps revealed a distinct seasonality in vector infestation. The highest indices were observed between epidemiological weeks 44 and 18, spanning November to April. During this period, the average TPI was 26%, the OPI was 86%, and the EDI was 137. Conversely, the period of lowest infestation occurred between epidemiological weeks 19 and 43, corresponding to May through October. During this time, the average indices were TPI: 16%, IPO: 72%, and IDO: 69. Figure 4 illustrates the transition between these periods of low and high infestation.

### Assembling Epidemiological and Entomological Data

**Figure 5.**
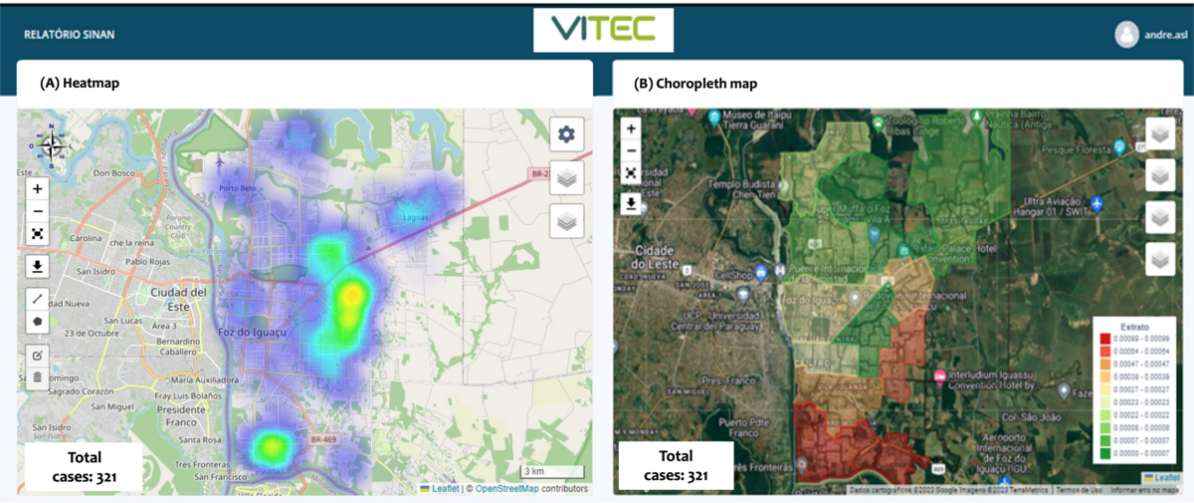
Dengue transmission risk maps for Foz do Iguaçu in the two first epidemiological weeks of 2023. (**A**) Heat map (kernel density estimation) showing the areas of Foz do Iguaçu with higher concentrations of dengue reports. (**B**) Choropleth map respecting the city subdivision in 12 areas of 8000‐12,000 dwellings each. The panel is composed of figures extracted from VITEC. VITEC: Vigilância Integrada com Tecnologia.

The VITEC enabled the integration of diverse data sources into a single database, encompassing field service routines, entomological data, and epidemiological data. This integration allowed managers to enhance their activity planning by consolidating georeferenced data collected by agents or imported from SINAN into tables, graphs, and maps. The primary tool used for decision-making was risk maps (Figure 5). The generated reports facilitated the monitoring of the spatial distribution of activities, staff performance, and program targets, as well as the production of quality and risk indicators in the form of indices and coefficients. Spatial analysis, through the integration of these indicators, aids in tracking the historical evolution of risk and outcomes and supports the integration of reports for the establishment of a virtual or physical situation room.

## Discussion

Despite the current widespread availability of technology and the critical importance of actions aimed at the prevention and control of arboviruses, the public sector continues to face significant challenges in adopting technological innovations for its practices. Nowadays, there are several modern strategies focused on adding innovative approaches to enhance surveillance and vector control [43,44]. Some of them directly target mosquito vectors, whereas others make use of genetically modifying the vector, their microbiome, symbionts, or insect-specific viruses [45-49]. Some of the improvements reported in the scientific literature are related to data gathering and management [50,51]. Data collection and tabulation in most tropical endemic areas within resource-limited countries still rely on archaic practices such as annotation in printed spreadsheets later stored in large warehouses [52]. The spatial information is handled manually and improvised with unscaled drawings representing the geographic area of interest, printed and pasted on a Styrofoam layer, and pinned the spatial information [52]. The adoption of a surveillance system would be helpful to modernize how the information is managed, by allowing a timely decision-making process to take place [44,50]. Given the varied levels of technical expertise among local technicians, any new tool must be both practical and user-friendly.

The implementation of VITEC has enabled more efficient and accurate diagnostics of local transmission risk, leading to a better understanding of operational activity patterns and risks. This efficiency provides more time for organizing services focused on prevention and outbreak control, thanks to the rapid data processing and relational capabilities of the system [13,51]. The reduction of rework in data importation and exportation has minimized errors, improved recording and analysis routines, and reduced the number of employees needed for data entry and further analysis. Additionally, VITEC has empowered health managers by reducing bureaucracy and facilitating leadership through the automated and timely generation of reports to support decision-making [52]. The system’s real-time, dynamic access to information allows local managers to promptly access field data, enabling immediate adjustments to operational activities and the implementation of preventive measures [13,29,30,53]. The mobile app facilitates agile and spatially accurate data collection, georeferenced in real time, which aids field personnel and allows managers to monitor dengue incidence in real time [34,52].

The traditional methods of planning and data collection for vector control activities, prevalent in most vector control services, have been effectively superseded by the implementation of softwares and electronic databases such as VITEC [54]. The VITEC system’s integrated web environment and mobile app have made planning field operations and generating service orders more dynamic and objective. Demands are transmitted to field applications for execution, and the data collected in the field are sent back to the web platform for real-time production of information in the form of maps, graphs, and tables. This process management approach has enhanced assertiveness, objectivity, and impartiality in evaluating results, such as monitoring agreed production targets per agent and team. The system also allows for precise tracking of the location and timing of service execution, thereby improving the evaluation of results associated with individual and collective operational activities.

Decentralizing the epidemiological surveillance process is essential for more effective decision-making [55]. Making data available across numerous health service sectors and accessible to health professionals involved in arbovirus prevention and control increases the likelihood of successful outcomes [12,19,20]. VITEC’s capability to import SINAN files daily and automatically produce maps and graphs of dengue cases and various disease indicators ensures precise spatial and temporal analysis. This capability supports targeted transmission control actions and active searches for symptomatic cases for diagnosis and follow-up care, aiming to prevent clinical deterioration.

The challenges of precariousness, improvisation, inaccuracy, and delays in data collection and availability for entomological surveillance that are well-known for arbovirus data have been addressed with the VITEC mobile app [56]. The app, equipped with a QR code reader for traps and barcode scanning for samples, ensures geographically and temporally accurate data from field-installed traps. Real-time data availability and entomological reporting provide managers with optimal decision-making tools to guide vector control services and prevent disease cases [57-59]. For example, by providing real-time monitoring of field traps and dengue report data using VITEC, information about the local epidemiological situation is immediately available for public health users. Our previous research in Foz do Iguaçu indicates that dengue cases tend to increase 15 to 30 days following a rise in mosquito infestation rates. Therefore, the timely availability of entomological information is crucial for successful prevention and control actions further—those with higher vulnerability [22,42]. After realizing an increase in entomological indexes, local public health managers would be able to check the regions within the city with higher mosquito collection and get a better response by directing further interventions to those areas.

VITEC has demonstrated its dynamic and flexible capacity to incorporate new technologies such as automated species identification sensors [60], drones [61], and predictive models based on machine learning techniques [62]. The system generates reports in the form of maps, tables, and graphs to represent vector infestation, disease epidemiology, and the progress of routine operational actions. These reports contribute to the creation of a “Situation Room,” displaying dashboards that can be monitored in real time via web access. The dashboards provide information for (1) entomological surveillance—indicators for eggs, larvae, and adults—and (2) epidemiological surveillance—mapping notified cases, endemic limits, age and gender distribution, and case distribution by notifying units.

Although the adoption of VITEC presents clear advantages over more traditional surveillance approaches, it also has some limitations. One of the most important limitations is that VITEC has been actually used in Brazil in 10 cities with distinct sizes and epidemiological statuses. This means that VITEC is under constant updation to incorporate new improvements into its code. One limitation VITEC may face is based on how cities are updating their entomological and epidemiological databases. VITEC is able to merge these data into a geographic information system to support local public health managers, but not updating raw data on a reasonable trend would jeopardize the ability of VITEC to support timely decisions. Despite the simplicity associated with using VITEC, an immediate limitation encountered is the cultural transition required in the short term, as the user needs to adapt to the existing routines in VITEC, where a digitalized routine is naturally different from a manual paper routine. Training and solidifying the concepts and routines require support materials, as well as hours for training and for dealing with doubts in the field. Another limitation is access to technological equipment such as computers, cell phones, or institutional tablets, as well as internet access.

In conclusion, several processes have been directly impacted by the use of VITEC, including the registration of employees, the structuring of work teams, the creation and updating of the city’s digital map (or importing an existing digital map), the registration of equipment and strategic supplies for surveillance routines, the opening and follow-up of monitoring and control requests, data collection by field agents, the generation of files to feed Sistema do Programa Nacional de Controle da Dengue, the importation of data from SINAN via file import, the customization of dashboards for viewing geoprocessed data and reports, and an environment for posting laboratory sample results.

## Supplementary material

10.2196/62759 Multimedia Appendix 1 Example of operational report based on one of the less productive health agents, who inspected an average of 6.57 Adultraps per working day. The name of the health agent and the address of each trap were omitted due to ethical reasons. The raw data presented are extracted from Vigilância Integrada com Tecnologia as a CSV file.

## Abbreviations

ADI: Adult Density Index
DENV: dengue virus
EDI: Egg Density Index
OPI: Ovitrap Positivity Index
SINAN: Information System for Notifiable Diseases
TPI: Trap Positivity Index
VITEC: Vigilância Integrada com Tecnologia
