# Supplementary material for: Development of an Integrated Surveillance System to Improve Preparedness for Arbovirus Outbreaks in a Dengue Endemic Setting: Descriptive Study
Source: JMIR Public Health Surveill. 2024 Nov 14;10:e62759. doi: 10.2196/62759 (PMC11611802; doi:10.2196/62759)
Supplement: Multimedia Appendix 1 [file publichealth-v10-e62759-s001.docx]

| Trap code | Latitude | Longitude | Day of installation | Time of installation | Inspection | Time of inspection | Days between inspections |
| --- | --- | --- | --- | --- | --- | --- | --- |
| 2523 | -254.947.594 | -545.646.135 | 06.05.24 | 11:02 | 20.05.24 | 11:07 | 14 |
| 2524 | -254.936.833 | -545.440.904 | 06.05.24 | 13:17 | 20.05.24 | 15:24 | 14 |
| 3250 | -255.032.616 | -54.546.601 | 06.05.24 | 13:17 | 22.05.24 | 11:45 | 16 |
| 2521 | -25.488.969 | -545.440.684 | 06.05.24 | 15:53 | 20.05.24 | 16:24 | 14 |
| 2566 | -254.911.005 | -54.540.831 | 06.05.24 | 16:08 | 20.05.24 | 14:38 | 14 |
| 2773 | -255.001.901 | -545.581.379 | 06.05.24 | 16:08 | 29.05.24 | - | 23 |
| 2505 | -254.908.887 | -545.439.816 | 06.05.24 | 16:08 | 29.05.24 | - | 23 |
| 2759 | -255.025.376 | -545.586.607 | 06.05.24 | 16:08 | 29.05.24 | - | 23 |
| 2765 | -254.999.939 | -545.617.551 | 06.05.24 | 17:44 | 20.05.24 | 10:18 | 14 |
| 2607 | -25.491.643 | -54.558.678 | 08.05.24 | 09:25 | 20.05.24 | 15:09 | 12 |
| 2742 | -254.972.215 | -545.585.941 | 08.05.24 | 09:25 | 20.05.24 | 11:52 | 12 |
| 2770 | -254.938.108 | -545.576.595 | 08.05.24 | 09:25 | 20.05.24 | 13:54 | 12 |
| 2758 | -254.975.871 | -545.623.443 | 08.05.24 | 09:25 | 20.05.24 | 12:04 | 12 |
| 2614 | -25.499.378 | -545.557.837 | 08.05.24 | 09:25 | 29.05.24 | - | 21 |
| 2600 | -254.974.407 | -545.551.924 | 08.05.24 | 13:35 | 22.05.24 | 10:26 | 14 |
| 2637 | -254.938.666 | -545.554.307 | 08.05.24 | 13:43 | 20.05.24 | 17:14 | 12 |
| 2632 | -254.917.239 | -545.516.663 | 08.05.24 | 13:43 | 22.05.24 | 17:49 | 14 |
| 2630 | -254.946.102 | -545.502.396 | 08.05.24 | 13:43 | 24.05.24 | 12:19 | 16 |
| 2526 | -254.914.637 | -545.468.464 | 08.05.24 | 13:43 | 29.05.24 | - | 21 |
| 2633 | -254.917.585 | -545.571.116 | 08.05.24 | 16:06 | 22.05.24 | 18:01 | 14 |
| 2625 | -254.940.033 | -545.470.713 | 10.05.24 | 08:41 | 22.05.24 | 10:13 | 12 |
| 2621 | -254.975.827 | -545.495.825 | 10.05.24 | 08:41 | 22.05.24 | 11:15 | 12 |
| 2628 | -255.029.017 | -545.499.288 | 10.05.24 | 09:42 | 22.05.24 | 09:37 | 12 |
| 3304 | -255.004.084 | -5.453.578 | 10.05.24 | 09:42 | 22.05.24 | 15:10 | 12 |
| 2629 | -255.006.125 | -545.529.044 | 10.05.24 | 09:42 | 29.05.24 | - | 19 |
| 3276 | -255.093.277 | -54.557.507 | 10.05.24 | 16:34 | 22.05.24 | 15:42 | 12 |
| 2763 | -255.002.357 | -545.649.898 | 16.05.24 | 09:45 | 20.05.24 | 10:32 | 4 |
| 2572 | -254.974.278 | -545.648.797 | 16.05.24 | 11:57 | 20.05.24 | 11:21 | 4 |
| 504 | -254.886.677 | -545.533.037 | 16.05.24 | 14:01 | 23.05.24 | 11:28 | 7 |
| 2673 | -254.874.238 | -54.555.193 | 16.05.24 | 14:01 | 23.05.24 | 11:18 | 7 |
| 1673 | -255.252.253 | -545.915.438 | 18.05.24 | 11:24 | 28.05.24 | 15:40 | 10 |
| 1809 | -255.256.234 | -545.898.154 | 18.05.24 | 11:24 | 28.05.24 | 09:29 | 10 |
| 1636 | -255.249.058 | -545.888.645 | 18.05.24 | 11:24 | 28.05.24 | 08:55 | 10 |
| 1197 | -25.525.099 | -545.924.647 | 18.05.24 | 11:24 | 28.05.24 | 16:02 | 10 |
| 1601 | -255.244.937 | -545.928.781 | 18.05.24 | 11:24 | 28.05.24 | 09:20 | 10 |
| 1621 | -255.236.931 | -545.890.887 | 18.05.24 | 11:24 | 28.05.24 | 09:40 | 10 |
| 2707 | -255.025.202 | -545.585.666 | 20.05.24 | 11:42 | 20.05.24 | 11:42 | 0 |
| 2623 | -254.914.565 | -545.471.301 | 20.05.24 | 16:54 | 20.05.24 | 16:54 | 0 |
| 3307 | -255.010.223 | -545.386.616 | 20.05.24 | 14:27 | 22.05.24 | 14:27 | 2 |
| 2598 | -255.067.691 | -54.550.226 | 20.05.24 | 14:05 | 24.05.24 | 14:05 | 4 |
